# Renoprotective Effect of a Chinese Herbal Formula, Qidan Dihuang Decoction, on Streptozotocin-Induced Diabetes in Rat

**DOI:** 10.1155/2018/7321086

**Published:** 2018-04-12

**Authors:** Z. J. Chen, F. Ma, X. M. Sun, X. S. Zhao, R. Luo

**Affiliations:** Department of Traditional Chinese Medicine, Nan Fang Southern Medical University, Guangzhou City, Guangdong, China

## Abstract

Qidan Dihuang decoction (QDD) is the latest development of Chinese medicine compound and mainly provides renal protection. The study presented was designed to evaluate the renoprotective effects of QDD on streptozotocin-induced diabetes and to explore the possible mechanisms of this action. We established a diabetes rat model. The condition of the rats was observed. The biochemistry indexes for diabetic rats were examined. Renal tissues were stained with HE, PAS, and Masson and we performed immunohistochemical staining for *α*-SMA and TGF-*β*. The proteins expressions of *α*-SMA, TGF-*β*, renin, and AT1 were detected by western blot. After treatment for 8 weeks, serum creatinine and 24 h proteinuria were significantly reduced in the rats which received losartan and Qidan Dihuang decoction while blood glucose, urine volume, blood urea nitrogen, and KW/BW did not improve. The pathology of renal tissue of rats treated with losartan and Qidan Dihuang decoction was inhibited. In addition, western blot showed that the expression of *α*-SMA, TGF-*β*, renin, and AT1 proteins was significantly decreased after receiving Qidan Dihuang decoction and losartan. Taken together, the results indicate that Qidan Dihuang decoction can improve the renal function and inhibit renal fibrosis of DN rat via modulating RAS system.

## 1. Background and Introduction

Diabetic mellitus is the most common metabolic disease and the fastest growing disease worldwide [1]. The global prevalence of diabetes mellitus is estimated to be 8.3% and continues to rise, particularly in developing countries [2]. Diabetic nephropathy (DN), a common complication of diabetes, is a progressive kidney disease which is characterized by nodular glomerulosclerosis. It is the leading cause for the development of end-stage renal disease (ESRD). Patients with diabetes and chronic kidney disease are at high risk of progression to ESRD, requiring maintenance dialysis of transplantation [3]. In addition, this condition has also become a concern from the economic point of view, due to healthcare costs [4].

Renal fibrosis, the final common manifestation of DN, is characterized by glomerulosclerosis and tubulointerstitial fibrosis [5]. The pathogenesis of renal fibrosis is characterized by an excessive accumulation and deposition of extracellular matrix (ECM) components [5]. Myofibroblasts are considered to be the important causes of DN glomerular sclerosis, which produce collagen and regulate connective tissue remodeling by combining the ECM. The role for myofibroblasts in organ fibrosis is speculated to be diverse and dynamic in the progression of disease. In the context of renal fibrosis, myofibroblasts persist, and the ECM continues to accumulate as a result of irreversibly tissue insult, which ultimately leads to ESRD [6]. Therefore, the myofibroblast is a major player in the onset and evolution of renal fibrosis and has been implicated in pathogenesis.

Although more than dozens of fibrogenic factors have been confirmed, transforming growth factor-*β* (TGF-*β*) and its downstream Smad signaling doubtlessly plays a central role among the fibrogenic factors. Upregulation of TGF-*β* is a universal finding in virtually every type of chronic kidney disease (CKD), either in animal models or in humans. It was previously reported that TGF-*β* can stimulate mesangial cells, interstitial fibroblasts, and tubular epithelial cells to undergo myofibroblastic activation or transition, to become matrix-producing fibrogenic cells. Conversely, inhibition of TGF-*β* by multiple strategies is able to prevent progressive loss of kidney function and suppresses renal fibrotic lesions.

Angiotensin II and high glucose are upstream inducer of TGF-*β* and their effect is integrated by the TGF-*β* induction [5]. The renin-angiotensin system (RAS) is involved in a variety of pathological processes involved in renal insult [7]. Previous studies suggested that the RAS contributes to the development and progression of kidney disease in diabetes [8]. The efficacy of the RAS blockade was demonstrated in patients at various stages of diabetic nephropathy with slowing of the progression of renal injury. However, although the RAS inhibitors are widely used, the prevalence of diabetic nephropathy continues to be high.

Traditional Chinese medicine (TCM) has a long history in the treatment of diabetes. Increasing numbers of studies are focusing on the efficacy of TCM on the treatment of diabetes and its complications [9]. QDD is a fresh herb compound composed of Radix Astragali (Huangqi), Radix Salvia Miltiorrhizae (Danshen), Radix Rehmanniae (Shengdi), Chinese yam (Shanyao), and Liquorice (Gancao) and developed via the internal medicine department of TCM of Southern Medical University which is based on the etiology and pathogenesis of TCM considered of DM, “Qi and Yin deficiency with blood stasis,” and combined with the previous literature reports and data analysis, of which*, Radix Astragali and Radix Salvia Miltiorrhizae, *the primary herbs of QDD, have been documented to improve renal fibrosis [10] and alleviate DM [11], which reminds us that Chinese herbal compound recipe consisting of the two herbs is possible to treat the renal fibrosis induced by the DN. Thus, we hypothesized that QDD can exhibit its renoprotection and fibrosis improved via inhibiting RAS activation. For the current study, we aimed at determining the effect of the Chinese herbal decoction QDD on renal function indications and protein expression of *α*-SMA, TGF-*β*_1_, renin, and angiotensin II type 1 receptor (AT_1_ receptor) in STZ-induced (streptozotocin-induced) diabetic rats.

## 2. Materials and Methods

### 2.1. Drug Preparation

QDD was composed of Huangqi, Danshen, Shengdi, Shanyao, and Gancaom, with a weight ratio of 6 : 3 : 3 : 3 : 1 and its dosage is as shown in Table 1. A total amount of 8.3 g of mixture was decocted for twice under reflux with double distilled water for 2 h and 1 h, respectively. The resultant decoction was centrifuged at 2000*g* for 15 min at 4°C. The resulting filtration was sequentially concentrated to a paste in a rotary evaporator. The final yield of power extract is 3 g/ml. The thick extract was diluted to proper concentration (1.5 g/ml) prior to administering losartan tablets (Cozaar 50 mg, batch number: J20140148) produced by Merck Sharp and Dohme Limited were given in 20 mg/kg and suspended in distilled water, resulting in a concentration of 3.6 mg/ml. The process was manipulated by the National Engineering and Research Center for Traditional Chinese Medicine under internationally certified good manufacturing practice guidelines.

### 2.2. Experimental Animals

Sprague-Dawley (SD) rats (male, weight 250–300 g) were obtained from the Animal Experiment Center of Southern Medical University, Guangzhou, China. The approval number of the Southern Medical University Animal Care and Use Committee is SCXK (Guangdong) 2011-0015. Animals were housed in a temperature- and humidity-controlled room with a 12 : 12 h light-dark cycle. All the rats were provided with rat normal pellet diet and water ad libitum. All experimental protocols were approved by the Southern Medical University Animal Care and Use Committee and procedures were carried out in accordance with National Institutes of Health Guidelines. After 1 week of assimilation and an overnight fast, 10 rats were kept as control group and the remaining rats were induced to diabetes by intravenous injection of streptozotocin (65 mg/kg; Sigma-Aldrich, St. Louis, MO, USA). The rats of control group received natrium citricum buffer solution with the same volume. Blood glucose levels were measured 3 and 5 days after streptozotocin injection using a hand-held glucometer (Roche ACCU-CHEK Performa, USA) by tail vein puncture blood sampling. The rats with blood glucose levels ≥ 16.7 mM on both days were defined as diabetic [12] and used in this study. In total, 49 rats were brought into the present study including 10 rats of control group (normal rats treated with vehicle) and 39 diabetic rats divided into three groups (*n* = 13 in each group): diabetes group (diabetes rats treated with vehicle); losartan group (diabetes rat treated with losartan, 20 mg/kg); and QDD group (treated with QDD).

### 2.3. Biochemical Analysis

Rats were housed in individual metabolic cages to record urine output and to collect urinary samples. Every two weeks after treatment with losartan or QDD, blood glucose was detected by glucometer. Every four weeks, blood was collected from the rat eye socket vein to determine blood urea nitrogen (BUN). Serum creatinine was tested at 8 weeks after treatment. Urine samples were collected with metabolism cage of rats for 24 h urinary protein (24U-Pro) concentration. All the tests were performed by commercially available kits (Scr-Test and BUN-Test, Nanjing Jiancheng Bioengineering Institution, China; Enhanced BCA Protein Assay Kit, Beyotime, China). At 8 weeks after treatment, under deep anaesthesia with urethane (1.2 g/kg, ip), the kidneys of rats were removed, weighted, and stored at −80°C for subsequent analysis.

### 2.4. Morphologic and Immunohistochemical Evaluation

A part of the kidney that was removed from each mouse was fixed in 10% formalin in phosphate buffer (pH 7.4). After fixation in formaldehyde, kidney tissues were embedded in paraffin and 4 *μ* thick slices were stained with the hematoxylin-eosin (HE), periodic acid-Schiff (PAS), and Masson methods. Light microscopy was used to semiquantitatively evaluate kidney sections. Sections were assessed using computer software. The collagen volume fraction was calculated as a percentage of the stained area of glomerular capsules within a field.

### 2.5. Histology and Immunohistochemical Staining

Paraffin-embedded mouse kidney sections (4 *μ*m thickness) were prepared by a routine procedure. Sections were subjected to hematoxylin-eosin (HE), periodic acid-Schiff (PAS), and Masson's trichrome staining (MTS) using a standard protocol for assessing collagen deposition and fibrotic lesions. Semiquantification of the tissue fibrotic lesions area was carried out by a computer-aided point-counting morphometric analysis (MetaMorph, Universal Imaging Co., Downingtown, PA). Immunohistochemical staining was performed using routine protocol. Paraffin-embedded sections were stained with rabbit polyclonal*α*-SMA antibody (ab616; Abcam Inc., Cambridge, MA) and anti-TGF*β* antibody (sc-9024, Santa Cruz Biotechnology) using the methods as described previously.

### 2.6. Western Blotting

Protein expression was analyzed by western blot analysis. The primary antibodies used were as follows: rabbit polyclonal anti-*α*-SMA antibody (Sigma-Aldrich) and mouse anti-glyceraldehyde 3-phosphate dehydrogenase (GAPDH) (BOSTER, Wuhan, China). The renal tissue was lysed in lysis regent and after 5 min centrifugation at 10,000 ×g, the supernatant was collected and protein levels were estimated by BCA protein assay kit (Beyotime). Protein (40 *μ*g) was loaded equally in each well on 8% polyacrylamide gels for gel-electrophoresis and then electransferred to polyvinylidene difluoride membranes. After blocking the blots for 1 h with 5% skimmed milk, the gels were incubated separately for 16 h with rabbit polyclonal anti-*α*-SMA antibody (1 : 1000 dilution), mouse anti-GAPDH (1 : 1000 dilution), rabbit polyclonal anti-TGF-*β*_1_ antibody (1 : 1000 dilution), rabbit polyclonal anti-AT_1_ antibody (1 : 1000 dilution), and rabbit polyclonal anti-Renin antibody (1 : 1000 dilution). A secondary antibody was conjugated with horseradish peroxidase-linked anti-rabbit antibody (1 : 5000, Santa Cruz Biotechnology)

### 2.7. Statistical Analysis

Data are expressed as the mean ± SEM. Variation between groups was analyzed by one-way analysis of variance (ANOVA) test and least significant difference (LSD) was measured using post hoc* multiple comparison *tests. The Statistical Software for Social Sciences (SPSS; 23) provided by IBM (USA) was used and a value of *P* < 0.05 was considered statistically significant.

## 3. Results

### 3.1. Effect of QDD on Blood Glucose in DN Rats

**Figure 1:**
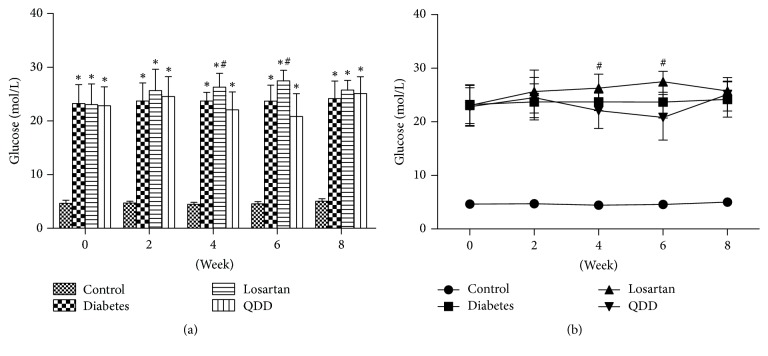
Effect of QDD on glucose of DN Rats. (a) Bar graph of glucose in four groups measured at 0, 2, 4, 6, and 8 weeks; (b) line graph of glucose in four groups measured at 0, 2, 4, 6, and 8 weeks. Data were analyzed by one-way ANOVA followed by the least significant difference. Data were shown as mean ± SEM, *n* = 10 (control group) or 13 (other groups);  ^*∗*^*P* < 0.05 versus control group;  ^#^*P* < 0.05 versus diabetes group.

The blood glucose of rats from the four groups at 0, 2, 4, 6, and 8 weeks is shown in Table 2. The blood glucose of rats in the DN group, the diabetes group, and the losartan group was markedly increased as compared with that in the control group at 0, 2, 4, 6, and 8 weeks. Compared with the model group, there was no significant increase in blood glucose between the losartan and QDD groups at 0 and 2 weeks. At 4 and 6 weeks, the blood glucose in the losartan group had significantly increased than that in the DN group. At 8 weeks after administration, the blood glucose of the rats in the losartan group and the QDD group was not significantly different compared to that in the model group, as shown in Figures 1(a) and 1(b).

### 3.2. Effect of QDD on Body Weight of DN Rats

**Figure 2:**
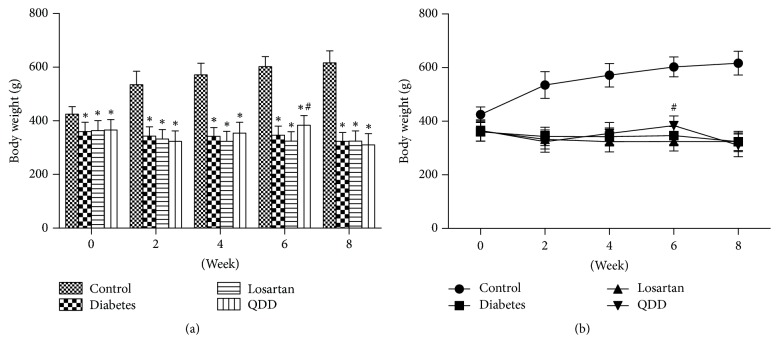
Effect of QDD on body weight of DN Rats. (a) Bar graph of body weight in four groups measured at 0, 2, 4, 6, and 8 weeks; (b) line graph of body weight in four groups measured at 0, 2, 4, 6, and 8 weeks. Data were analyzed by one-way ANOVA followed by the least significant difference. Data were shown as mean ± SEM, *n* = 10 (control group) or 13 (other groups);  ^*∗*^*P* < 0.05 versus control group;  ^#^*P* < 0.05 versus diabetes group.

The body weight of rats in the four groups at 0, 2, 4, 6, and 8 weeks as shown in Table 3. The body weights of rats in diabetes group, losartan group, and QDD group were significantly lower than that in control group at 0, 2, and 4 weeks. Compared with the diabetes group, there was no significant difference in the body weight of the rats in the losartan group and QDD group at 0, 2, 4, 6, and 8 weeks, as shown in Figures 2(a) and 2(b).

### 3.3. Effect of QDD on Urine Volume in DN Rats

**Figure 3:**
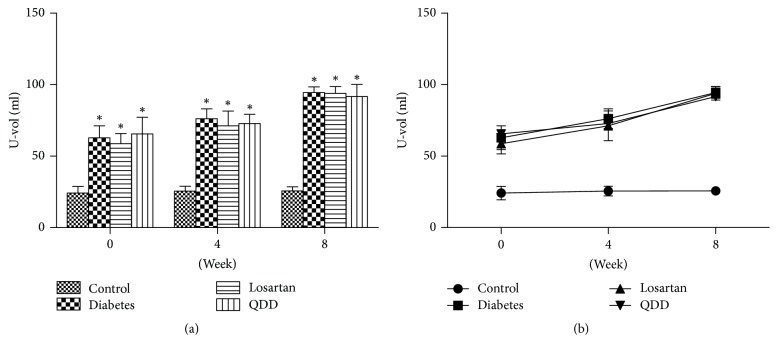
Effect of QDD on U-vol of DN rats. (a) Bar graph of U-vol in four groups measured at 0, 2, 4, 6, and 8 weeks; (b) line graph of U-vol in four groups measured at 0, 2, 4, 6, and 8 weeks. Data were analyzed by one-way ANOVA followed by the least significant difference. Data were shown as mean ± SEM, *n* = 10 (control group) or 13 (other groups);  ^*∗*^*P* < 0.05 versus control group;  ^#^*P* < 0.05 versus diabetes group.

The U-vol of rats in the four groups at 0, 4, and 8 weeks is shown in Table 4. The U-vol of the QDD group, the diabetes group, and the losartan group was significantly increased than that in the normal group at 0, 4, and 8 weeks. However, there was no significant difference in the U-vol among the QDD group, the losartan group, and the diabetes group, as shown in Figures 3(a) and 3(b).

### 3.4. Effect of QDD on the Scr in Four Groups at 8 Weeks after Administration

**Figure 4:**
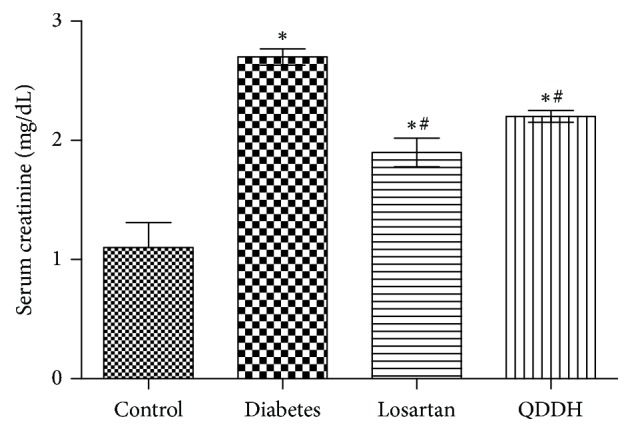
Effect of QDD on Scr of DN rats. Bar graph of Scr in four groups measured at 8 weeks; data were analyzed by one-way ANOVA followed by the least significant difference. Data were shown as mean ± SEM, *n* = 10 (control group) or 13 (other groups);  ^*∗*^*P* < 0.05 versus control group;  ^#^*P* < 0.05 versus diabetes group.

The Scr of four groups of rats at 8 weeks is shown in Table 5. Scr in QDD rats, diabetes rats, and losartan rats were higher than those in control group. Compared with the diabetes group, the Scr of QDD group and losartan group decreased significantly after 8 weeks of administration, as shown in Figure 4.

### 3.5. Effect of QDD on the BUN in Four Groups at 0, 4, and 8 Weeks after Administration

**Figure 5:**
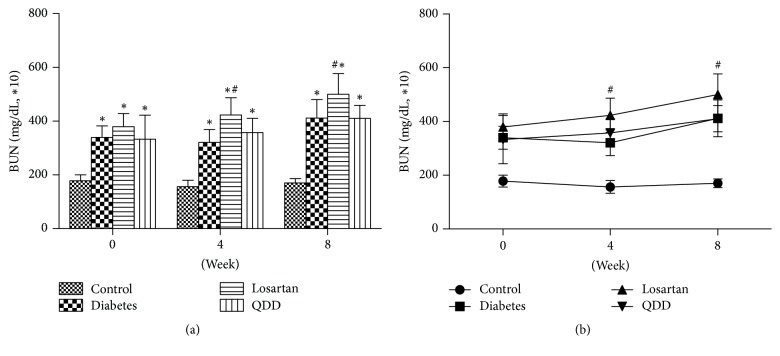
Effect of QDD on BUN of DN rats. (a) Bar graph of BUN in four groups measured at 0, 2, 4, 6, and 8 weeks; (b) line graph of BUN in four groups measured at 0, 2, 4, 6, and 8 weeks. Data were analyzed by one-way ANOVA followed by the least significant difference. Data were shown as mean ± SEM, *n* = 10 (control group) or 13 (other groups);  ^*∗*^*P* < 0.05 versus control group;  ^#^*P* < 0.05 versus diabetes group.

The BUN of the four groups rats at 0, 4, and 8 weeks as shown in Table 6. The BUN in the diabetes rats, losartan rats, and the QDD rats were significantly increased compared to those in the control group at 0, 4, and 8 weeks after the administration. There was no significant difference among QDD, losartan, and diabetes groups. At 4 and 8 weeks after administration, the BUN of rats in QDD group had no significant difference. However, the BUN of rats in losartan group and diabetes group had significant increased, as shown in Figures 5(a) and 5(b).

### 3.6. Effect of QDD on the 24 hU-pro in Four Groups at 0, 4, and 8 Weeks after Administration

**Figure 6:**
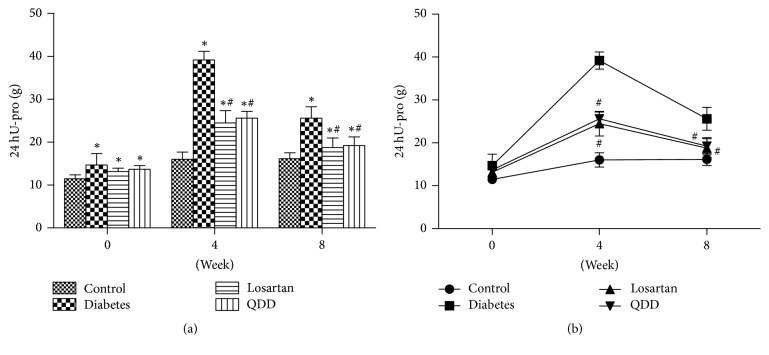
Effect of QDD on 24 HU-pro of DN rats. (a) Bar graph of 24 HU-pro in four groups measured at 0, 2, 4, 6, and 8 weeks; (b) line graph of 24 HU-pro in four groups measured at 0, 2, 4, 6, and 8 weeks. Data were analyzed by one-way ANOVA followed by the least significant difference. Data were shown as mean ± SEM, *n* = 10 (control group) or 13 (other groups);  ^*∗*^*P* < 0.05 versus control group;  ^#^*P* < 0.05 versus diabetes group.

The 24 hU-pro in the four groups of rats at 0, 4, and 8 weeks is shown in Table 7. 24 hU-Pro in QDD group, diabetes group, and losartan group were higher than those in control group. After 4 and 8 weeks of administration, the 24 hU-Pro of the rats in QDD group and losartan group was significantly lower than that in the diabetes group, as shown in Figures 6(a) and 6(b).

### 3.7. Effect of QDD on KW/BW in DN Rats

**Figure 7:**
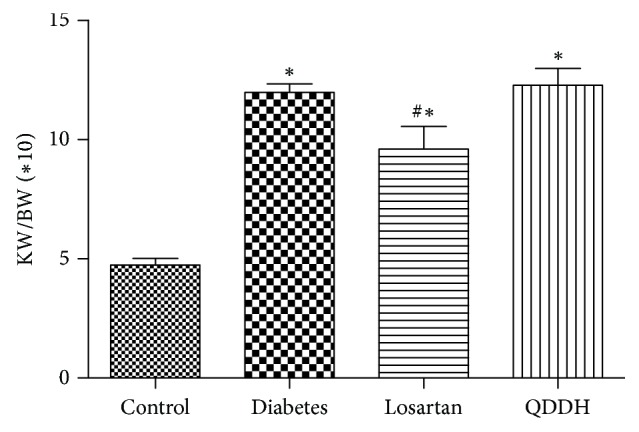
Effect of QDD on KW/BW of DN rats. Data were shown as mean ± SEM, *n* = 10 (control group) or 13 (other groups);  ^*∗*^*P* < 0.05 versus control group;  ^#^*P* < 0.05 versus diabetes group.

The KW/BW in the four groups rats at 0, 4, and 8 weeks as shown in Table 8. KW/BW values of rats in the QDD group, diabetes group, and losartan group were significantly higher than those in the control group after 8 weeks of administration. Compared with the diabetes group, there was no significant difference in KW/BW between the QDD and losartan group, while the KW/BW value of the losartan group was significantly different from that of the diabetes group, as shown in Figure 7.

### 3.8. Effect of QDD on Renal Pathology in DN Rats

**Figure 8:**
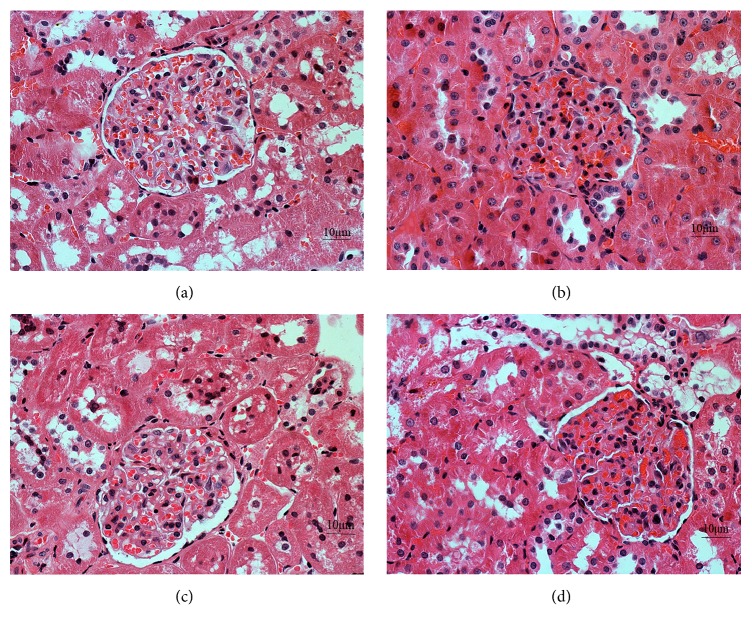
Effect of QDD on the renal section with HE staining in four groups (400x). (a) Control group; (b) diabetes group; (c) losartan group; and (d) QDD group. Scale bar, 10 *μ*m.

**Figure 9:**
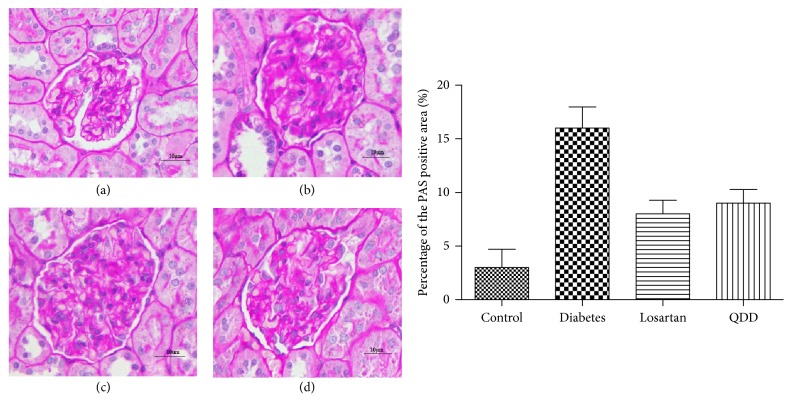
Effect of QDD on the renal section with PAS staining in four groups (400x). (a) Control group; (b) diabetes group; (c) losartan group; and (d) QDD group. Scale bar, 10 *μ*m.

**Figure 10:**
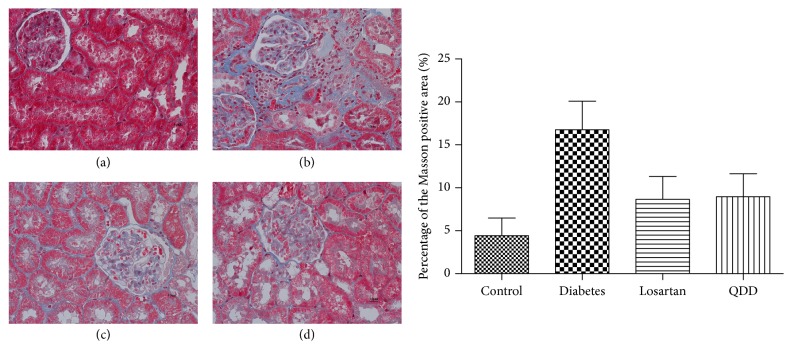
Effect of QDD on the renal section with Masson staining in four groups (400x). (a) Control group; (b) diabetes group; (c) losartan group; and (d) QDD group. Scale bar, 10 *μ*m.

Glomerular and tubular structures were examined by HE, PAS, and Masson's stains, respectively. As shown in Figures 8, 9, and 10, the glomerular and renal tubular structure of kidneys in rats of control group. However, there were inflammatory cells infiltrated, impressive mesangial expansion, and basement membranes thickened in rats of DN group. Moreover, there was significant collagen deposition and fibrosis in the tubule interstitium of rats in DN group.

### 3.9. Effect of QDD on Immunochemistry in DN Rat

**Figure 11:**
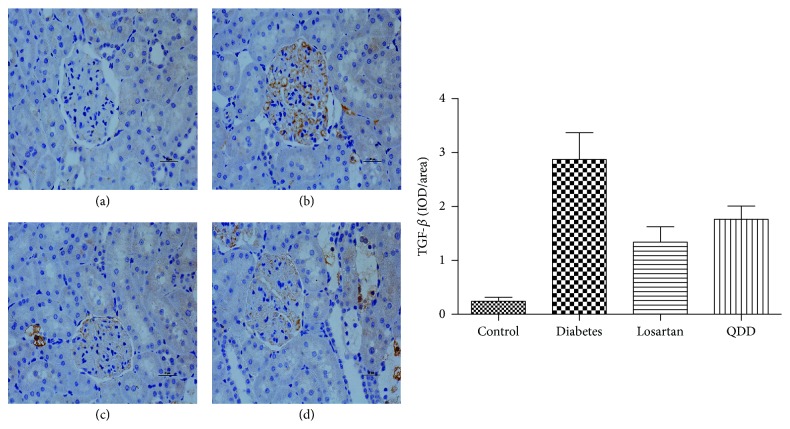
Effect of QDD on the renal section with immunochemistry staining of TGF-*β* in four groups (400x). (a) Control group; (b) diabetes group; (c) losartan group; and (d) QDD group. Scale bar, 10 *μ*m.

**Figure 12:**
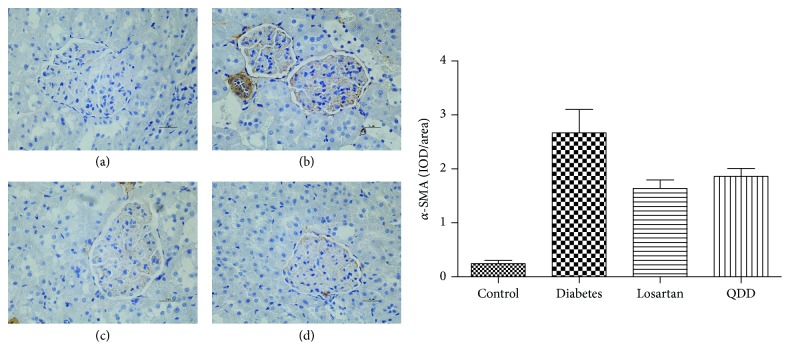
Effect of QDD on the renal section with immunochemistry staining of *α*-SMA in four groups (400x). (a) Control group; (b) diabetes group; (c) losartan group; and (d) QDD group. Scale bar, 10 *μ*m.

TGF-*β* and *α*-SMA are important proteins associated with ECM accumulation. In this stud, diabetic rats showed a higher expression of TGF-*β* and *α*-SMA in the renal cortex compared with normal rats (*P* < 0.05). Administration of QDD and losartan significantly decreased the high expression of TGF-*β* and *α*-SMA induced by diabetes in the glomerular, as shown in Figures 11 and 12.

### 3.10. Effect of QDD on Protein Expression in DN Rats

**Figure 13:**
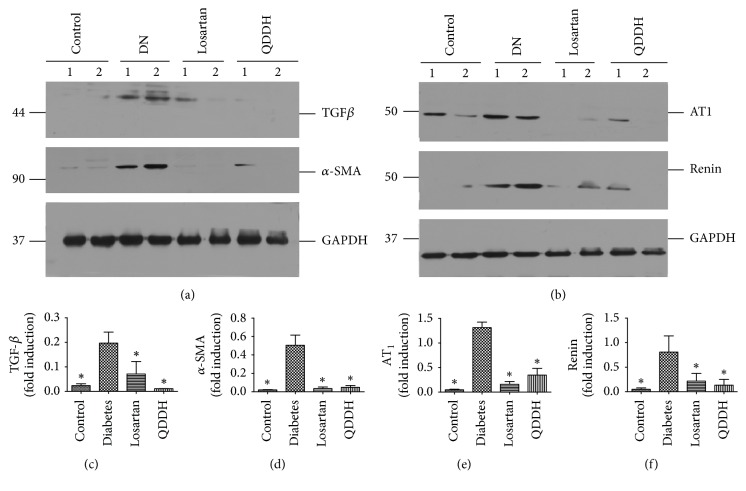
Effect of QDD on renal protein expression. Losartan or QDD remarkably reduced AT_1_, renin, TGF-*β*_1_, and *α*-SMA expression. (a) Images of TGF*β* and *α*-SMA relative protein level measured by western blot, normalized to GAPDH. (b) Images of AT_1_ and renin relative protein level measured by western blot, normalized to GAPDH. (c–f) Graphic presentations of the relative abundances of TGF*β*, *α*-SMA, AT_1_, and renin in different groups as indicated.  ^*∗*^*P* < 0.05 versus diabetes alone.

To investigate the effect of QDD on the myofibroblasts, we determined the protein expression of *α*-SAM. As shown in Figure 4, *α*-SAM protein expression was remarkably upregulated in the diabetic rats and inhibited by both of QDD and losartan. To further study the role of TGF-*β*_1_ in the diabetes rats treated by QDD, the TGF-*β*_1_ protein expression was detected and increased significantly in diabetic rats and reduced by QDD and losartan. To further investigate whether the RAS system played important roles in the course of renal function improving and renal fibrosis inhibition via QDD, we examined the protein expression of AT_1_ and renin, respectively. Despite the impressive upregulation of both proteins in diabetic rats, they are decreased via losartan and QDD treatment, as shown in Figure 13.

## 4. Discussion

DM and its renal complication have been a serious global health problem. DN is one of the major “microvascular” complications of diabetes. End-stage renal disease in diabetes has increased despite ACEI and angiotensin receptor blocker (ARB) use [8]. The therapeutic effects for diabetes and its complications are limited due to unavailability of effective medications. TCM has demonstrated a good practice in the treatment of diabetes mellitus and its complications [9, 10]. The present study was aimed to investigate the effect of Chinese herbal decoction QDD, which consists of* Radix Astragali, Radix Salvia Miltiorrhizae, Radix Rehmanniae, Chinese yam, and Liquorice*, on diabetes and its renal complication. There were lots of impressed researches involved in the primary herbs of QDD to improve renal function by reducing Scr and proteinuria [10, 11].* Radix Astragali* is derived from the dried root of* Astragalus membranaceus *which has been used as Chinese medicine for over hundreds of years [11].* Radix Astragali* traditionally was used to strengthen superficial resistance and promote growth of new tissue. Numerous studies have reported that* Astragali Radix* shows positive results on the renal function in diabetes. Nagasaka et al. [13] reported on four cases of chronic renal failure effectively treated with Astragali Radix. In their study,* Radix Astragali* had a useful effect on the Cr level, but it could not change BUN, proteinuria, and anemia. This study suggests that* Radix Astragali* is a useful agent in the treatment of GRF.* Radix Rehmanniae* is the common herb for treating nephropathy as well. Lau et al. [11] confirmed that the synergistic interaction between* Radix Astragali* and* Radix Rehmanniae* could promote diabetic wound healing. In their study, herbal formula NF3 comprising* Radix Astragali* and* Radix Astragali* in the ratio of 2 : 1 was found effective in enhancing diabetic wound healing in rats through the actions of tissue regeneration, angiogenesis promotion, and inflammation inhibition.* Radix Astragali*,* Radix Rehmanniae,* and NF3 did not affect the body weights of rats. Chinese yam belongs to the Dioscoreaceae family and has been widely used to promote health and also used in Asian traditional medicine for the treatment of diabetes. Studies from Go et al. [14] show that Chinese yam has antidiabetic effects by modulating antioxidant activities, lipid profiles, and promoting the release of GLP-1, thereby improving the function of *β*-cells to maintain normal insulin and glucose levels. Liquorice is an important traditional herbal and it is believed that it has a role in reconciling various herbs. Modern pharmacological studies found the antidiabetic effect. The main water-soluble constituent of the root of liquorice is glycyrrhizin. Sen et al. [15] reported that glycyrrhizin is quite effective against hyperglycaemia, hyperlipidaemia, and associated oxidative stress and may be a potential therapeutic agent for diabetes treatment. As in present study, QDD significantly reduced Scr and 24 hU-pro which shows the Chinese herb compound could improve the renal function of diabetic rats. Nevertheless, the remarkable improvements occurred without blood glucose, body weight, urine volume, and BUN melioration. The results are consistent to several previous researches.

The pathology of diabetic nephropathy includes hypertrophy of various cell types of the glomerular and tubular basement membranes and expansion of tubule-interstitial and mesangial compartments [16]. Numbers of studies demonstrated that* Radix Astragali, Radix Rehmanniae, and Radix Salvia Miltiorrhiza *could improve renal structure in diabetic rats [10, 11, 17]. In this study, QDD, as the losartan, could inhibit inflammatory invasion and reduced glomerular glycogen deposition and matrix accumulation. The results were consistent to the previous studies [10, 11, 17].

After kidney injury, fibroblasts differentiate into contractile and secretory myofibroblasts that contribute to tissue repair but that can severely impair kidney function when contraction and extracellular matrix (ECM) protein secretion become excessive to lead to renal fibrosis [6]. Therefore, the inhibition of excessive myofibroblasts could ameliorate renal fibrosis. In this study, QDD and losartan impressively reduced *α*-SMA protein expression, which suggest that QDD could inhibit the excessive myofibroblasts, and immunochemistry staining further corroborated the results. A number of previous studies have demonstrated that TGF-*β* is the key mediator in CKD associated with progressive renal fibrosis [5]. Considerable evidence revealed that TGF-*β* is substantially upregulated in the injured kidney on both patients and animal disease models [18, 19]. As a result in this study, TGF-*β* protein expression was significantly reduced by QDD and losartan, which suggest the QDD is likely to moderate TGF-*β*_1_ to inhibit excessive myofibroblasts. Previous reports confirmed that the intrarenal RAS would be activated in those with renal disease, such as diabetic nephropathy [20]. In the current study, we showed that AT_1_ receptor and renin protein expression were downregulated by QDD and losartan. The above results suggested that QDD may play a role in renoprotection and antifibrosis in DN and it maybe works through regulating RAS.

## 5. Conclusion

In summary, QDD exerted renoprotection effects, including an attenuation of 24 hU-pro and Scr, and inhibited the renal expression of *α*-SMA and TGF*β*_1_ as well as AT_1_ and renin in STZ-induced diabetic rats. Our findings might therefore provide novel choice into the renoprotection conferred by QDD against renal dysfunction and fibrosis in diabetes.

## Figures and Tables

**Table 1:** The components of QDD and dosage used.

Component	Clinical dosage (g/60 kg)	Rat dosage (g/kg)
*Radix Astragali*	30	3.15
*Radix Salvia Miltiorrhizae*	15	1.56
*Radix Rehmanniae*	15	1.56
*Chinese yam *	15	1.56
*Liquorice *	5	0.5

**Table 2:** Effect of QDD on the blood glucose in four groups at 0, 2, 4, 6, and 8 weeks after administration.

Group	Blood glucose of rats in four groups at 0, 2, 4, 6, and 8 weeks after administration				
	0 weeks	2 weeks	4 weeks	6 weeks	8 weeks
Control	4.65 ± 0.59	4.71 ± 0.33	4.46 ± 0.39	4.57 ± 0.41	5.02 ± 0.49
Diabetes	23.23 ± 3.55^*∗*^	23.72 ± 3.36^*∗*^	23.71 ± 1.63^*∗*^	23.69 ± 2.97^*∗*^	24.17 ± 3.28^*∗*^
Losartan	23.04 ± 3.87^*∗*^	25.64 ± 4.00^*∗*^	26.28 ± 2.60^*∗*#^	27.48 ± 1.95^*∗*#^	25.75 ± 1.82^*∗*^
QDD	22.83 ± 3.51^*∗*^	24.53 ± 3.74^*∗*^	22.08 ± 3.32^*∗*^	20.83 ± 4.24^*∗*^	25.12 ± 3.12^*∗*^

Data are reported as means ± SD.  ^*∗*^*P* < 0.05 versus control;  ^#^*P* < 0.05 versus diabetes.

**Table 3:** Effect of QDD on the body weight in four groups at 0, 2, 4, 6, and 8 weeks after administration.

Group	Body weight (g) of rats in four groups at 0, 2, 4, 6, and 8 weeks after administration				
	0 weeks	2 weeks	4 weeks	6 weeks	8 weeks
Control	424.70 ± 27.99	534.90 ± 50.12	571.40 ± 43.60	602.60 ± 37.24	616.40 ± 44.27
Diabetes	360.51 ± 34.18^*∗*^	343.33 ± 34.40^*∗*^	342.00 ± 32.73^*∗*^	346.42 ± 33.63^*∗*^	323.42 ± 32.91^*∗*^
Losartan	363.49 ± 37.82^*∗*^	332.16 ± 35.81^*∗*^	323.50 ± 37.73^*∗*^	324.33 ± 35.15^*∗*^	324.17 ± 38.04^*∗*^
QDD	365.38 ± 39.81^*∗*^	323.48 ± 38.83^*∗*^	354.31 ± 40.80^*∗*^	383.38 ± 36.28^*∗*#^	310.00 ± 42.30^*∗*^

Data are reported as means ± SD.  ^*∗*^*P* < 0.05 versus control;  ^#^*P* < 0.05 versus diabetes.

**Table 4:** Effect of QDD on the U-vol (ml) in four groups at 0, 4, and 8 weeks after administration.

Group	U-vol of rats in four groups at 0, 4, and 8 weeks after administration		
	0 week s	4 weeks	8 weeks
Control	24.15 ± 4.72	25.52 ± 3.36	25.65 ± 2.86
Diabetes	62.85 ± 8.29^*∗*^	76.22 ± 6.86^*∗*^	94.44 ± 3.98^*∗*^
Losartan	58.71 ± 7.09^*∗*^	71.16 ± 10.36^*∗*^	93.92 ± 4.73^*∗*^
QDD	65.58 ± 11.63^*∗*^	72.75 ± 6.51^*∗*^	91.66 ± 8.56^*∗*^

Data are reported as means ± SD.  ^*∗*^*P* < 0.05 versus control;  ^#^*P* < 0.05 versus diabetes.

**Table 5:** Effect of QDD on the Scr in four groups at 8 weeks after administration.

Group	Scr (mg/dL)
Control	1.1 ± 0.21
Diabetes	2.7 ± 0.07^*∗*^
Losartan	1.9 ± 0.12^*∗*#^
QDD	2.2 ± 0.05^*∗*#^

Data are reported as means ± SD.  ^*∗*^*P* < 0.05 versus control;  ^#^*P* < 0.05 versus diabetes.

**Table 6:** Effect of QDD on the BUN in four groups at 0, 4, and 8 weeks after administration.

Group	BUN (mg/dL*∗*10) of rats in four groups at 0, 4, and 8 weeks after administration		
	0 weeks	4 weeks	8 weeks
Control	178.2 ± 22.1	156.5 ± 23.5	170.3 ± 15.9
Diabetes	339.5 ± 42.6^*∗*^	321.0 ± 48.1^*∗*^	411.9 ± 68.5^*∗*^
Losartan	332.9 ± 48.9^*∗*^	423.0 ± 63.7^*∗*#^	500.4 ± 76.8^*∗*#^
QDD	379.5 ± 89.7^*∗*^	357.6 ± 52.4^*∗*^	410.4 ± 48.9^*∗*^

Data are reported as means ± SD.  ^*∗*^*P* < 0.05 versus control;  ^#^*P* < 0.05 versus diabetes.

**Table 7:** Effect of QDD on the 24hU-pro in four groups at 0, 4, and 8 weeks after administration.

Group	24hU-pro (g) of rats in four groups at 0, 4, and 8 weeks after administration		
	0 weeks	4 weeks	8 weeks
Control	11.48 ± 0.92	16.13 ± 1.66	16.02 ± 1.38
Diabetes	14.69 ± 2.65^*∗*^	25.60 ± 1.99^*∗*^	39.18 ± 2.65^*∗*^
Losartan	13.52 ± 0.77^*∗*^	18.78 ± 2.87^*∗*#^	24.49 ± 2.20^*∗*#^
QDD	13.68 ± 0.90^*∗*^	19.21 ± 1.56^*∗*#^	25.61 ± 2.03^*∗*#^

Data are reported as means ± SD.  ^*∗*^*P* < 0.05 versus control;  ^#^*P* < 0.05 versus diabetes.

**Table 8:** Effect of QDD on the KW/BW in four groups at 8 weeks after administration.

Group	KW/BW/×10^3^
Control	4.74 ± 0.90
Diabetes	11.98 ± 1.25^*∗*^
Losartan	9.61 ± 3.29^*∗*#^
QDD	12.28 ± 2.61^*∗*^

Data are reported as means ± SD.  ^*∗*^*P* < 0.05 versus control;  ^#^*P* < 0.05 versus diabetes.
